# Lake microbiome and trophy fluctuations of the ancient hemp rettery

**DOI:** 10.1038/s41598-022-12761-w

**Published:** 2022-05-25

**Authors:** Olga Iwańska, Przemysław Latoch, Magdalena Suchora, Irena A. Pidek, Miłosz Huber, Iwona Bubak, Natalia Kopik, Mariia Kovalenko, Michał Gąsiorowski, Jean-Paul Armache, Agata L. Starosta

**Affiliations:** 1 ECOTECH-Complex and Institute of Biological Sciences, Maria Curie-Sklodowska University, Lublin, Poland; 2 Institute of Biochemistry and Biophysics, Polish Academy of Science, Warsaw, Poland; 3 Polish-Japanese Academy of Information Technology, Warsaw, Poland; 4 Institute of Earth and Environmental Sciences, Maria Curie-Sklodowska University, Lublin, Poland; 5 ECOTECH-Complex, Maria Curie-Sklodowska University, Lublin, Poland; 6 Department of Hydrology, Institute of Geography, University of Gdansk, Gdansk, Poland; 7 Institute of Geological Sciences Polish Academy of Sciences, Warsaw, Poland; 8 Department of Biochemistry and Molecular Biology and the Huck Institutes of Life Sciences, Pennsylvania State University, University Park, PA USA

**Keywords:** Limnology, Metagenomics

## Abstract

Lake sediments not only store the long-term ecological information including pollen and microfossils but are also a source of sedimentary DNA (*sed*DNA). Here, by the combination of traditional multi-proxy paleolimnological methods with the whole-metagenome shotgun-sequencing of *sed*DNA we were able to paint a comprehensive picture of the fluctuations in trophy and bacterial diversity and metabolism of a small temperate lake in response to hemp retting, across the past 2000 years. Hemp retting (HR), a key step in hemp fibre production, was historically carried out in freshwater reservoirs and had a negative impact on the lake ecosystems. In Lake Slone, we identified two HR events, during the late stage of the Roman and Early Medieval periods and correlated these to the increased trophy and imbalanced lake microbiome. The metagenomic analyses showed a higher abundance of Chloroflexi, Planctomycetes and Bacteroidetes and a functional shift towards anaerobic metabolism, including degradation of complex biopolymers such as pectin and cellulose, during HR episodes. The lake eutrophication during HR was linked to the allochthonous, rather than autochthonous carbon supply—hemp straws. We also showed that the identification of HR based on the palynological analysis of hemp pollen may be inconclusive and we suggest the employment of the fibre count analysis as an additional and independent proxy.

## Introduction

The lake ecosystems are sensitive to external impacts, such as climate change and anthropogenic factors^1^. The responses to stressors applied either to the lake itself or to the catchment are recorded in lake sediments constituting well-established sources of historical archives, which can be investigated via a number of paleolimnological proxies^2,3^. In addition to the classical paleolimnological analyses, the lake sediments have also recently become subjects of the next-generation sequencing (NGS) studies resulting in amplicon and shotgun metagenomic records^1^. Lake sediments constitute anaerobic and relatively inert conditions and are therefore fairly adequate for the preservation of the sedimentary DNA (*sed*DNA)^4^. The microbiome fluctuations over long periods of time in response to the environmental and anthropogenic stressors can thus be reconstructed from the lake sediments and can provide new and complementary information to the classical paleolimnological analyses^5^. As a result, the field of metagenomics in paleoecological research is new and rapidly developing with an increasing number of studies applying shotgun sequencing^5,6^ and combining multi-proxy paleo-environmental data with the NGS data^1,7,8^.

The use and processing of hemp (*Cannabis sativa* L.) in Europe dates back to the Bronze age, where it spread during the Roman times and reached its peak in the early Middle Ages^9,10^. Hemp retting (HR) is a central step of hemp fibre production which was historically carried out in freshwater reservoirs like ponds, lakes, or bogs (water-retting). HR on the fields (dew-retting) and in the artificial water reservoirs emerged later, probably as an attempt to avoid pollution of freshwater reservoirs^11^. Indeed, HR has a high environmental impact due to the biological nature of the process. During water-retting the straws are submerged in water and soluble materials including polysaccharides dissolve, ensuring nutrients for the developing retting microorganisms which are critical to the process^12^. The microbiome of a hemp rettery depends on the levels of dissolved oxygen, type of degraded biomass^13,14^, and also on the geochemical and physical factors which influence the initial reservoir flora^15^.

**Figure 1:**
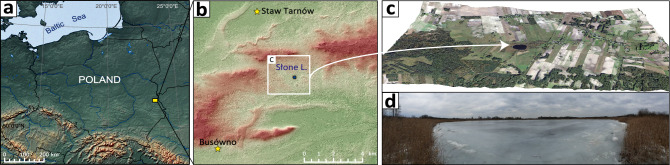
Localization of Lake Slone. (**a**) Lake Slone (51°18′16″N, 23°21′55″E) is located in SE Poland within the Chełm Hills Region, a part of Volhyn Polesie^18^. (**b**) Topography of the northern part of Chełm Hills Region showing the locations of the closest Early Medieval hillforts—Busówno and Staw Tarnów marked with yellow stars—in the vicinity of Lake Slone. In the Middle Ages, at least four settlement units functioned within the 10 km radius from the study site, with Busówno hillfort being the closest and the largest among them^19^. The results of geoarchaeological studies of the medieval settlements in the region have been well-described^20^ however, earlier anthropogenic activity in SE Poland dating back to the Roman period and the Migration Period has not been fully characterised. During the Roman period, the Przeworsk and Wielbark cultures can be located to the region of SE Poland, which then migrated towards the south and west and were replaced by the first Slavs around the sixth century^21^. The first records of the settlement in Busówno date back to the seventh century and the archaeological evidence suggests that the hillfort functioned until the fourteenth century and was still used, but to a lesser extent, until the seventeenth century^19^. (**c**) Nowadays, Lake Slone is a small, shallow (3.4 ha, 8.0 m), eutrophic water body (Carlson Trophic State Index = 44–58) of high ecological status^17^. The lake has a catchment area of 5.5 km^2^ with a predominant agricultural land use (arable lands, meadows and wetlands), whereas build-up areas within the catchment do not exceed 2%. (**d**) Ice-covered Lake Slone during sediment sampling, January 2019.

Our previous investigations of Lake Slone (LS) (Fig. 1a), located in SE Poland, revealed that it was used as a hemp rettery in the early Middle Ages^16^, most probably by the nearby settlers (Fig. 1b). The relatively large catchment, together with the small lake area and volume results in a high eutrophication risk^17^ and as a consequence, LS reacts readily to the environmental changes in its catchment (Fig. 1c,d). The recurrent nature of HR events during the last 2000 years of LS history allowed us to investigate the long-term response of the lake microbiome to the changing anthropopressure, especially in the context of anthropogenically induced trophy fluctuations.

In this highly interdisciplinary study, we used multi-proxy paleolimnological data and combined it with the metagenome shotgun sequencing of the 1 m long LS sediment core collected in 2019 (LS-C19) to identify the historical periods of HR in the lake and examine their effects on lake trophy and bacterial microbiome fluctuations, in the context of environmental changes. Our study shed light onto the microbiome of a hemp rettery versus a recovered lake as a consequence of varying anthropopressure during the preindustrial period, by providing a detailed analysis of the microbial taxonomy and functionality based on the *sed*DNA analysis.

## Results

### Paleo-environmental analysis

**Figure 2:**
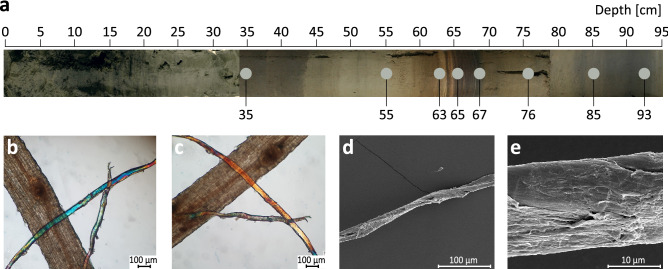
Lake Slone sediment core and hemp fibres. (**a**) Photo of the LS-0 sediment core (composite core sampled parallel to LS-C19 core with Instorf-type of sediment sampler), exhibiting distinct lithological changes of the sediment with an approximate location of the layers selected for metagenomic DNA isolation (material taken from LS-C19 core). (**b**,**c**) Polarised light microscopy images of hemp fibres extracted from a layer at 65 cm depth of the LS-C19 core. Results of the modified Herzog test^22^ to identify hemp bast fibres show the characteristic colour change from blue to yellow upon sample stage rotation by 90° which would be expected of hemp fibres, (**d**) Scanning Electron Microscopy (SEM) image of a hemp bast fibre collected from the layer at 65 cm depth from LS-C19 at the magnification × 500, (**e**) SEM image of a hemp bast fibre at the magnification × 5000.

The LS-C19 core comprises gyttja type of sediments with varied colouring and a distinguishable 5.5 cm thick laminated layer (62.5–68 cm from the sediment surface), containing macroscopically visible plant fibres, which were shown to be hemp fibres—remains of HR (Fig. 2).

**Figure 3:**
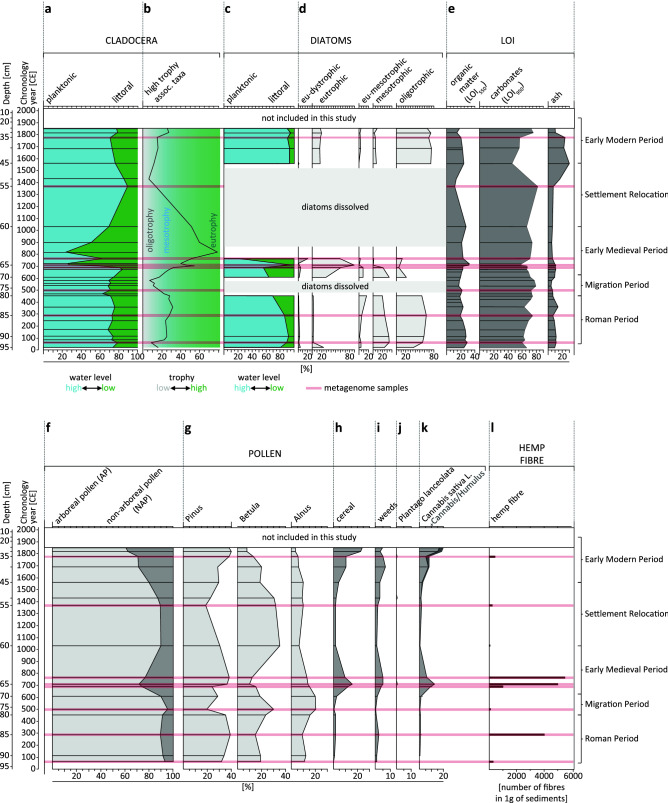
Paleo-environmental indicators. The age-depth correlation of the LS-C19 with paleo-environmental indicators: (**a**) Cladocera-based water level inference based on the proportion of planktonic to littoral taxa (Supp Table 1, Supp Fig. 3) in LS-C19 sediments; (**b**) Cladocera-based trophy inference based on the proportion of high trophy indicator taxa in Chydoridae family (Supp Table 1, Supp Fig. 3); (**c**) Diatom-based water level inference based on the proportion of planktonic to littoral taxa in LS-C19 sediments (Supp Table 2, Supp Fig. 4); (**d**) Diatom-based trophy inference shown as proportion of diatom taxa indicative to oligo-, meso-, eu- and oligotrophic status of water (Supp Table 2, Supp Fig. 4); (**e**) proportion of the sediment components based on loss on ignition (LOI) measurements at different temperatures—organic matter (550 °C), carbonate (950 °C) and ash (non-carbonate mineral residue) in LS-C19 sediments. Sediment composition of LS-C19 is dominated by the carbonate component (46–85%) with organic matter ranging from 11 to 32%, whereas the non-carbonate mineral matter ranges from 3 to 30% (Supp Data 1); (**f**) proportion of arboreal pollen (tree sum) to non-arboreal plants pollen, indicator of the landscape openness; (**g**) selected tree pollen curves; (**h**) sum of the cereal pollen (includes the pollen of *Secale cereale*, *Triticum* sp., *Linum usitatissimum*, Cerealia undiff., *Fagopyrum* sp., and *Cannabis sativa* L.); (**i**) sum of the weeds pollen (includes segetal and ruderal weeds *Centaurea cyanus*, *Convolvulus arvensis*, *Artemisia* sp., Chenopodiaceae, *Urtica*, *Brassicaceae*, *Rumex acetosella* t., *Spergula arvensis*, *Scleranthus annuus*); (**j**) *Plantago lanceolata* pollen sum (indicator of pastoral activity); (**k**) *Cannabis sativa* L. pollen and *Cannabis sativa*/*Humulus* type pollen sum (when unequivocal distinction of the grain type was impossible); (**l**) fibre count (absolute number) per 1 g of sediments, with threshold level for hemp retting practices (400 fibres/1 g); layers which *sedDNA* was extracted from are marked in red.

We used Cladocera and diatoms together with the loss on ignition analyses to investigate lake trophy, and palynological analysis to evaluate the intensity of anthropogenic activity. This, together with the composite age-depth model (Supp Figs. 1–4; Supp Tables 1, 2), allowed us to distinguish six phases in the lake development, correlated to the archaeological periods: (I) the Roman Period; (II) the Migration Period; (III) the Early Middle Ages; (IV) Settlement Relocation; (V) the Early Modern Period, and (VI) the Contemporary Times (Fig. 3). The Contemporary Time period was not a part of this study as it falls into the Great Acceleration period^23^.

During the Roman Period (93–80 cm, I–III CE) LS was relatively deep and alkaline, with a well-developed open-water zone (Fig. 3a,c), as shown by the presence of planktonic taxa of Cladocera and diatoms (*Lindavia* and *Cyclotella*), and dominance of alkaliphilous (*Lindavia ocellata* and *L. radiosa*), alkalibionthic (*Stephanodiscus parvus*) and neutrophilic (*Lindavia comensis* and *Cyclotella cyclopuncta*) groups of diatoms (Supp Figs. 3, 4; Supp Tables 1, 2). The paleoecological proxies imply the oligo-/mesotrophy level, however, at the depth 86–81 cm a slight elevation of the trophy was noticed, recorded as the increase and composition change of Cladocera (Fig. 3b, Supp Fig. 3, Supp Table 1). The palynological analysis revealed a minor anthropogenic influence involving deforestation (altered ratio of the arboreal pollen, AP, to non-arboreal pollen, NAP), presence of segetal weeds and agricultural plants, and sporadic occurrence of wheat (*Triticum t.*) and cereals (Cerealia undiff.) (Fig. 3f,h,i). In samples LS-85 and LS-80, corresponding to depths of 85 and 80 cm, we identified hemp pollen—0.69% (1.32% including *Cannabis/Humulus*) and 0.66% (0.86%) of the total pollen sum respectively—together with hemp fibres, suggesting LS was occasionally used as a hemp rettery as early as in the Roman Period (Fig. 3k,l).

The Migration Period (80–69 cm, IV–VII CE) was characterised by the relieved anthropogenic influences and forest regeneration (Fig. 3f,g), accompanied by a decrease in depth and lake trophy. The increase in benthic diatoms points to an extension of the euphotic zone (Fig. 3d). The improvement of benthic oxygen availability is evidenced by an increase in the abundance of Cladocera benthic taxa and a decrease in the littoral species (Fig. 3a,b; Supp Figs. 3, 4; Supp Tables 1, 2). A decrease in the NAP values including all cereals and *C. sativa*, and an increase in the AP values, further indicate a diminished anthropogenic influence (Fig. 3f,h,i,k). The fibre count in layers corresponding to this period was very low suggesting no HR practices (Fig. 3l).

The Early Middle Ages (69–60 cm, VII-XI CE) were the most distinguishable in terms of the ecological changes phase of the lake development with the corresponding layers covering the lamination zone (62.5–68.0 cm) (Fig. 2). The planktonic Cladocera and diatoms, dominant in the layers 69–67 cm, were gradually being replaced with the benthic species indicative of trophy and pH rise, also shown by the dominance of alkaliphilous (*Ulnaria contracta, Stephanodiscus medius, Gomphonema truncatum* and *G. olivaceum*) and alkalibiontic (*Stephanodiscus parvus*) diatoms (Supp Fig. 4; Supp Table 2). A drastic decrease in the Cladocera planktonic taxa proportion in the layers 66–63 cm suggests lake shallowing, whereas the increase of *Alona guttata*, *Alonella excisa* and *Kurzia lattisima* points to an elevated supply of humic substances (Fig. 3b). In the top layers (62–60 cm) the lake trophy was still high, however, HR activity probably ceased, as suggested by the palynological data and very low fibre count (50 fibres/g of sediment) (Fig. 3b,l; Supp Figs. 3, 4; Supp Tables 1, 2). The high anthropogenic activity in the area during the Early Middle Ages is evidenced by high (11.5–30.2%) NAP values indicative of arable fields, pastures and a significant degree of deforestation, as well as intensive agriculture of cereals (*Secale cereale* and *Triticum* t.) and hemp (Fig. 3f,h,i,j,k). The fibre count in layers 63 and 65 cm was the highest (up to 5500 fibres/g) suggesting the most intensive HR practices in LS in the Early Middle Ages (Fig. 3l).

During the Settlement Relocation (60–45 cm, XII-XVII CE), the NAP values consisting of both segetal weeds and crops decreased significantly (Fig. 3h,i). Such decrease also included hemp pollen (Fig. 3k). Together with the low values of fibre count, these reflect cessation of *Cannabis* cultivation and retting in the area (Fig. 3l) which strongly suggests settlement relocation, as supported by the archeological literature^19^. The absence of anthropopressure resulted in a clear oligotrophication and an increase in the water level indicated by the Cladocera-based inference (Fig. 3a,b). At 60–55 cm diatoms frustules were dissolved, however, after their reappearance at 45 cm, diatom composition confirmed the interpretation of Cladocera data suggesting oligotrophy and increased water level (Fig. 3c,d). Moreover, diatom composition at 45 cm points to a drop in the pH value to ≤ 7 (Supp Fig. 4).

The Early Modern Period (45–25 cm, XVIII–XIX CE) of the LS history is characterised by the returning anthropopressure evidenced by deforestation (decrease in AP values) and the increasing NAP values (crops, segetal and ruderal weeds) (Fig. 3f,h,i,j). Interestingly, we found high levels of hemp pollen, however, low fibre count suggests hemp cultivation but no HR in the lake (Fig. 3k,l). This is reflected in the lake trophy which remained at the oligo-mesotrophy with the elevated water level, as evidenced by the higher abundance of planktonic Cladocera and diatoms, compared to the benthic taxa (Fig. 3b,d).

### Shotgun metagenomics

Based on the paleo-environmental analysis, we selected eight layers for shotgun NGS. Samples were prepared in duplicates and originated from depths of 93 and 85 cm (Roman period), 76 cm (Migration period), 67, 65 and 63 cm (Early Middle Ages), 55 cm (Settlement Relocation) and 35 cm (Early Modern period). Samples nomenclature corresponds to the lake’s name (LS), extraction depth (as listed above) and duplicate number (1 or 2).

#### Taxonomic diversity

**Figure 4:**
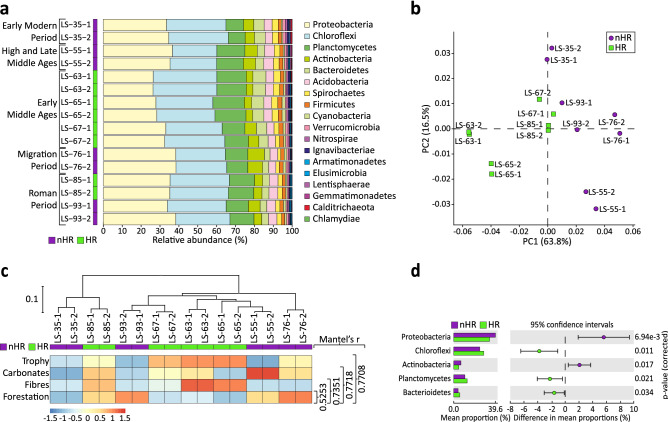
Taxonomic analysis of the microbiomes in the hemp retting (‘HR’) vs no hemp retting (‘nHR’) groups. (**a**) Taxonomical classification of the bacterial communities from Lake Slone. Relative abundances are presented at the phylum level. Samples from the periods of hemp retting (‘HR’) are coded in green, samples from the no hemp retting (‘nHR’) group are in purple. (**b**) Principal Component Analysis plot of the taxonomical structures of all samples in two groups—‘HR’ (green) vs ‘nHR’ (purple); (**c**) Hierarchical clustering (unweighted pair group method with arithmetic mean, UPGMA) based on taxonomy Bray–Curtis dissimilarity matrix (MEGAN). Samples from the two groups are colour coded—the ‘HR’ group in green and ‘nHR’ in purple. Heat map of the normalised values of the selected environmental factors showing the highest correlation to the taxonomic composition of the microbial communities, calculated by Mantel test (bioenv and mantel functions in vegan package), values of r-statistic indicated on the right, p < 0.01; (**d**) Extended error bar plot showing significant differences (p < 0.05) in the taxa abundances at the phylum level for ‘HR’ (green) vs ‘nHR’ (purple) groups, calculated with two-sided Welch’s *t* test (STAMP).

The taxonomical classification of the 16 samples resulted in 70% of the sequences assigned to taxonomic ranks (Supp Fig. 5a). The most dominant group was Bacteria (77–84%), followed by Archaea (13–20%), Eukaryota (0.5–3%) and Viruses (1–2%) (Supp Fig. 5b). The most abundant bacterial phyla in all samples were *Proteobacteria* (26.4–38.4%) and *Chloroflexi* (23.5–33.9%), followed by *Planctomycetes* (8.8–16.5%), *Bacteroidetes* (2.9–7.0%), *Actinobacteria* (3.5–8.7%), *Acidobacteria* (3.8–5.2%), *Spirochaetes* (1.6–3.4%), *Firmicutes* (1.6–2.2%) and *Verrucomicrobia* (0.6–1.4%) which in total account for 94.2–97.0% of the total abundance in each sample (Fig. 4a). The remaining ten phyla range between 0.0–1.0% per sample and account for the total of 3.0–5.8% abundance of bacterial communities.

Based on the hemp retting activity in LS we categorized the samples into the ‘no hemp retting’ group (‘nHR’ including 8 samples: LS-93-1/2, LS-76-1/2, LS-55-1/2, and LS-35-1/2) and ‘hemp retting’ group (‘HR’ including 8 samples: LS-85-1/2, LS-67-1/2, LS-65-1/2, LS-63-1/2) (Fig. 4b). The PCA revealed a clear grouping of the two groups: samples LS-65-1/2 and LS-63-1/2 (intensive lake usage as a rettery) clustered together and opposite to the LS-76-1/2 and LS-55-1/2 (settlement emptiness). LS-85-1/2 and LS-67-1/2 (moderate levels of HR) clustered together and in the middle section of the PCA plot and could thus represent the transitional state of the lake microbiome. Interestingly, LS-93-1/2 and LS-35-1/2—no HR but known anthropogenic presence—were also located in the middle section of the PCA plot. This may suggest shifts in the lake microbiome depending on the degree of anthropopressure. Likewise, in the UPGMA tree the samples originating from periods of no anthropogenic influence (LS-76-1/2, LS-55-1/2) clustered together and distant to the remaining layers. Samples corresponding to the intensive anthropogenic impact (LS-65-1/2, LS-63-1/2) were grouped together however, constituted a part of a more complex cluster including LS-35-1/2, LS-67-1/2, LS-85-1/2 and LS-93-1/2 (Fig. 4c). PERMANOVA analysis indeed showed a difference between the two groups of microbial communities (‘HR’ vs ‘nHR’, F = 6.2209, p < 0.01).

We further examined the correlation between the microbial communities and the investigated environmental factors (Supp Table 3). Four factors affected the microbiome the most and accounted for 77% of community dissimilarities. These included fibre count, forestation, carbonate content and trophy (p < 0.01) (Fig. 4c). The first two describe the anthropogenic impact on the lake microbiome—fibre count serves as a proxy for the intensity of HR whereas forestation (AP values) describes forest regeneration and the absence of human activities. Carbonate content and lake trophy independently describe physico-chemical characteristics including pH, oxygen concentration of the benthic zone and amount of organic carbon.

The taxonomic composition of the microbial communities varied notably depending on the usage of LS as a hemp rettery. We found significant differences in the abundance of five bacterial phyla (Fig. 4d). In the ‘HR’ group, three phyla were more abundant: Chloroflexi with the average abundance at 30.5 ± 1.5% (p < 0.01) and a difference in mean proportions (DM) between the two groups at − 3.9%, followed by Planctomycetes (13.8 ± 1.6%, p < 0.05, DM − 2.4%) and Bacteroidetes (5.5 ± 1.2%, p < 0.05, DM − 1.4%) (Supp Data 3a). In the ‘nHR’ group we identified two significantly overrepresented bacterial phyla, including Proteobacteria (38.0 ± 2.0%, p < 0.01, DM 5.6%) and Actinobacteria (6.4 ± 1.5%, p < 0.05, DM 2.0%). To further investigate the taxonomic changes, we identified significant differences in the relative abundances in 8 classes/orders in the ‘nHR’ group and 6 classes/orders in the ‘HR’ group (Supp Fig. 6). DM values were small (DM < 1%) for all groups, except Dehalococcoidia (DM − 1.05%; Supp Data 3b), demonstrating that bacterial community changes were subtle yet consistent. This suggests fluency in the process of adaptation to the new growth conditions rather than radical changes to the community composition.

#### Functional analysis

**Figure 5:**
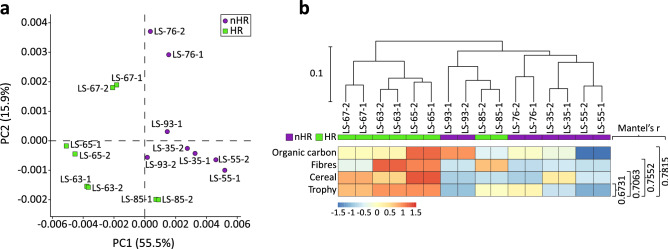
Functional analysis of the microbiomes in the hemp retting (‘HR’) vs no hemp retting (‘nHR’) groups. (**a**) Principal Component Analysis plot based on complete functional profiles of the samples in two groups—‘HR’ (green) vs ‘nHR’ (purple); (**b**) Hierarchical clustering (UPGMA) based on Bray–Curtis dissimilarity matrix of the complete functional profiles of all samples in the ‘HR’ (green) vs ‘nHR’ (purple) groups. Heat map of the normalised values of the selected environmental factors showing best correlation with the functional profiles, calculated by Mantel test (bioenv and mantel functions in vegan package), values of r-statistic indicated on the right, p < 0.01.

Data assembly and coding sequence prediction were followed by annotation and KEGG Orthology (KO) assignment (Supp Data 2b,c). The PCA performed on all KOs showed a similar ordination of the functional profiles compared to that of the taxonomical groups (Fig. 5a). Samples corresponding to the extensive HR activity (LS-63-1/2, LS-65-1/2) clustered together and distal to the centre of the PCA plot, and LS-67-1/2 (beginning of HR in the Early Middle Ages) clustered close by. Samples LS-85-1/2 (Roman episode of HR) were positioned closer to the middle part of the PCA plot. In the ‘nHR’ group, LS-35-1/2, LS-55-1/2 and LS-93-1/2 clustered together, and samples LS-76-1/2 (Migration Period) further away, closer to the middle section of the PCA plot. Interestingly, LS-35-1/2 and LS-55-1/2 clustered together on the functional PCA plot, in contrast to the taxonomy PCA plot, showing a degree of functional redundancy. A similar grouping was revealed by the UPGMA tree (Fig. 5b). Here, the functional structures of the microbiomes from LS-67, LS-65 and LS-63 (most intense HR) were grouped together and separate to the other samples. LS-76-1/2, LS-55-1/2, LS-35-1/2 clustered together and samples corresponding to the Roman Period (LS-93-1/2, LS-85-1/2) created a separate group, however, situated in a more complex cluster including samples from the ‘nHR’ group. PERMANOVA analysis of the functional profiles including all KOs revealed a statistically significant difference between the two groups (‘HR’ vs ‘nHR’, F = 6.0242, p < 0.01) and the functional and taxonomic compositions showed 76% relatedness (Mantel test, R^2^ = 0.7632, p < 0.01). The functional diversity of all samples (all identified KOs) was best described by four independent environmental factors that together contributed to 78% data similarity (p < 0.01). These included trophy, agriculture of cereals, fibre count and organic carbon (Fig. 5b). The cereal agriculture and fibre count describe the anthropogenic impact on the lake, both direct (HR) and indirect (cereal cultivation in the lake vicinity). Estimation of the organic carbon content (LOI_550_) provides information on both autochthonous and allochthonous carbon that has not been mineralized during sedimentation. Therefore, it is a proxy of lake productivity, catchment supply and mineralization conditions^24^.

#### Metabolic potential of the sediment microbiome

In order to determine differences in bacterial metabolism, we reconstructed the KEGG pathways from differentially abundant KOs using the MinPath tool^25^. 17.6% of all KOs were found in different proportions between the two groups (p < 0.05) (Supp Data 4), which resulted in 67 differentially abundant KEGG_level3_ pathways in the ‘HR’ group, and 91 pathways in the ‘nHR’ group (Supp Data 5). PERMANOVA analysis of the KOs classified to metabolism showed a moderate difference between the two groups (‘HR’ vs ‘nHR’, F = 5.7496, p < 0.01). Using the pathway reconstruction analysis, we further investigated nitrogen, sulphur and carbon metabolic pathways. We also calculated the genetic potential of the microbial communities towards different types of carbon metabolism using selected marker genes^26^ (Supp Table 4).

**Figure 6:**
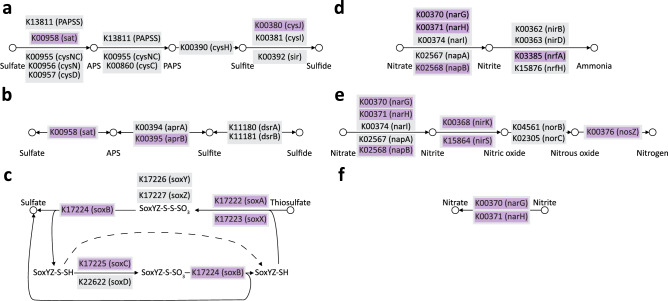
Schematics of the selected sulfur and nitrogen KEGG metabolic pathways. Pathways were inferred using the MinPath tool based on the overrepresented KEGG Orthologs (KOs), which were identified in the two-sided Welch’s *t* test (p < 0.05) in STAMP. KOs significantly overrepresented (p < 0.05) in the no hemp retting (‘nHR’) group are shown in purple boxes. KOs included in the KEGG pathway, but not significantly overrepresented (p > 0.05) are shown in grey boxes. (**a**) Assimilatory sulfate reduction, (**b**) Dissimilatory sulfate reduction and oxidation. (**c)** SOX system, (**d**) Dissimilatory nitrate reduction, (**e**) Denitrification, (**f**) Nitrification.

Sulfur metabolism was more pronounced in the ‘nHR’ group. Both dissimilatory and assimilatory sulfate reduction pathways were partially reconstructed, together with an almost complete sulfur oxidation pathway (Fig. 6a–c). No sulfur metabolism pathway was reconstructed based on the overrepresented reads originating from the ‘HR’ group. The inferred sulfur oxidation pathway contained reads classified mainly to Betaproteobacteria and Actinomycetes taxa (p < 0.05), while Deltaproteobacteria, Gammaproteobacteria and Betaproteobacteria were mostly responsible for sulfate reduction (p < 0.05). Likewise, reads classified to KOs describing nitrogen metabolism were more abundant in the ‘nHR’ group. Both dissimilatory nitrogen reduction and denitrification pathways were almost completely reconstructed. This was due to the overrepresentation of nitrate and nitrite reductases together with nitrous oxide reductase in the ‘nHR’ group. The nitrification pathway was partially reconstructed due to the higher abundance of nitrite reductases in the ‘nHR’ group (Fig. 6d–f). These were shown to be carried out mostly by Betaproteobacteria, Deltaproteobacteria and Gammaproteobacteria (p < 0.05).

**Figure 7:**
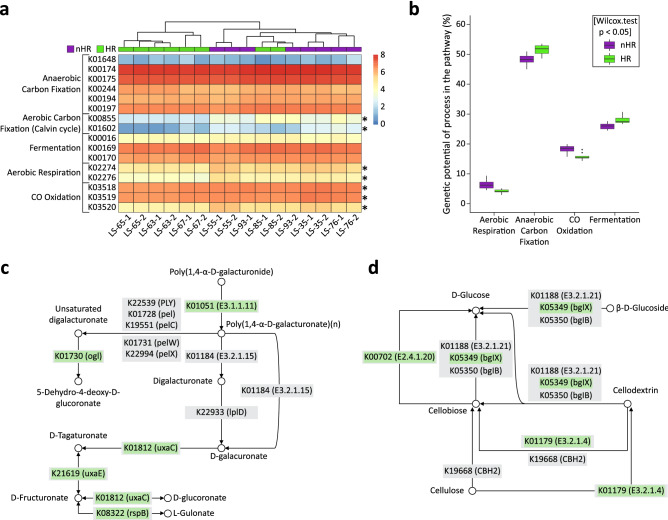
Carbon metabolism potential of the microbial communities in the ‘HR’ vs ‘nHR’ groups. (**a**) Heat map representing functional clustering of the selected KOs involved in different pathways of the carbon cycle^26^ (Supp Table 4) based on normalised relative abundances (log(TPM+1)) for all samples in the ‘HR’ (green) and ‘nHR’ (purple) groups. Differentially abundant KOs are marked with asterisks (Boruta, maxRuns = 9999, mean importance > 4). Hierarchical clustering (UPGMA) is based on the Bray–Curtis dissimilarity matrix for the selected KOs. (**b**) Box-and-whiskers plot of carbon metabolism potential calculated according to Lauro et al*.*^26^ based on relative abundances of the selected KOs in the ‘HR’ group (green) and ‘nHR’ group (purple): anaerobic carbon fixation—(K01648 + K00174 + K00175 + K00244 + K00194 + K00197)/6, aerobic carbon fixation—(K00855 + K01602)/2, fermentation—(K00016 + K00169 + K00170)/3, aerobic respiration—(K02274 + K02276)/2, CO oxidation—(K03518 + K03519 + K03520)/3. Significance of differences between group potentials tested with Wilcoxon test (p < 0.05). (**c**) Schematic of the inferred pectin hydrolysis pathway (fragment of KEGG 00040 Pentose and glucuronate interconversions pathway) based on the overrepresented KOs identified in two-sided Welch’s *t* test (p < 0.05). KOs overrepresented in the ‘HR’ group are shown in green boxes, KOs involved in the pathways, but not significantly overrepresented (p > 0.05) are shown in grey boxes. (**d**) Schematic of the inferred cellulose hydrolysis pathway (fragment of KEGG 00500 Starch and sucrose metabolism pathway) based on the overrepresented KOs identified in two-sided Welch’s *t* test (p < 0.05). KOs overexpressed in the ‘HR’ group are shown in green boxes. KOs involved in the pathways, but not significantly overrepresented (p > 0.05) are shown in grey boxes.

The most differentially abundant metabolic pathways in the ‘HR’ group were classified to carbon metabolism. Based on selected marker genes^26^ (Supp Table 4) we constructed the UPGMA tree which showed two distinct clusters. The first cluster included samples corresponding to the most intense HR activity in LS (LS-67-1/2, LS-65-1/2 and LS-63-1/2) and the second included samples from the ‘nHR’ group, as well as LS-85-1/2. We also identified marker genes which were differentially abundant between the two groups (p < 0.05) (Fig. 7a). Based on the calculated metabolic potential^26^, the most prevalent type of carbon metabolism in both groups was anaerobic carbon fixation, whereas aerobic respiration showed the lowest metabolic potential. Still, anaerobic carbon metabolism including fermentation and anaerobic carbon fixation was more pronounced in the ‘HR’ group (Wilcoxon test, p < 0.05) (Fig. 7b). Indeed, upon pathway reconstruction analysis we found the reductive Acetyl-CoA pathway and reductive citric acid cycle (TCA) to be partially inferred based on the overrepresented KOs in the ‘HR’ group. In the ‘nHR’ group the microbial community showed higher potential for CO oxidation and aerobic respiration (Fig. 7b). The marker genes for CO oxidation are not included in KEGG pathways which rendered pathway reconstruction analysis unfeasible. However, upon pathway reconstruction for aerobic respiration, several bacterial cytochrome complexes were shown to be highly abundant in the ‘nHR’ group. As the main source of organic carbon during HR episodes originated from hemp stems, we further investigated complex plant polysaccharides degradation pathways. Pectin and cellulose hydrolysis pathways were nearly completely reconstructed based on the differentially abundant reads (Fig. 7c,d). Pectin hydrolysing enzymes including pectin esterases (K01051) and pectate lyases (K01728, K19551 and K01731) were more abundant in the ‘HR’ group, however, for the latter the difference was not statistically significant (p < 0.1) (Fig. 7c). Interestingly, the number of reads mapped to endo-polygalacturonases (K01184), another important pectin-hydrolysing enzyme, was rather low and very similar in both groups. Also, no reads were classified to exo-polygalacturonases (K22933). This may suggest that hydrolysis of 1,4 glycosidic bonds between galacturonic acid residues could have been carried out by fungal enzymes and bacteria employed an alternative mechanism of pectin breakdown—eliminative cleavage of 1,4-α-d-galacturonan. The overrepresented pectin hydrolysing enzymes in the ‘HR’ group were classified mostly to Planctomycetes, Bacteroidetes, Chloroflexi and Clostridia. The remaining enzymes involved in pectin hydrolysis as well as the related conversions of galacturonate and glucuronate including glucuronate isomerase (K01812), tagaturonate epimerase (K21619), l-gulonate 5-dehydrogenase (K08322) and oligogalacturonide lyase (K01730) were also significantly more abundant in the ‘HR’ group (Fig. 7c). Although during HR cellulose hydrolysis is an unwanted by-product of microbial activity, a vast number of microorganisms are capable of cellulose degradation. We inferred a complete cellulose hydrolysis pathway based on the overrepresented reads from the ‘HR’ group (Fig. 7d), including endoglucanases (K01179), β-glucosidases (K05349) and cellobiose phosphorylases (K00702). Interestingly, as was the case for pectin hydrolysis, the number of reads assigned to exoglucanases (K19668) was relatively low and similar in both groups. Again, this may suggest participation of fungal enzymes in the cellulolytic processes. Based on the taxonomic classification of the overrepresented reads, cellulose degradation in the ‘HR’ group was carried out predominantly by Chloroflexi, Planctomycetes, Bacteroidetes, Acidobacteria, and to a smaller extent by Clostridia.

## Discussion

Based on the paleo-environmental data we identified increased anthropogenic activity in the lake vicinity during the late stage of the Roman Period, Early Middle Ages and Early Modern Period, and a markedly decreased anthropogenic influence during the Migration Period and High and Late Middle Ages. The cannabis type pollen records aligned with other palynological indicators of anthropopressure, however, did not correspond fully with the fibre count data. Typically, the determination of hemp water-retting has been based on the pollen records^10,27,28^. However, the use of cannabis type pollen percentages as determinants of HR has been discussed and different percentage cut-offs have been suggested^11,28^. Additional proxies for confirming water-retting of hemp have been proposed, including lithological changes, diatoms or algae^29,30^. The presence of phytophilic Chironomid taxa as potential indicators of HR in stagnant water was also discussed^11^. Hemp fibres were previously identified in the laminated lake sediments originating from the seasonal changes in biomass production and mineralisation and were reported as remains of hemp soaking^31^. Since lamination layers containing fibrous material were present in LS-C19, we employed fibre count as a proxy for HR and an estimate of the process intensity. Application of such approach revealed discrepancies between the hemp pollen and fibre count—we identified relatively low levels of *Cannabis* type pollen (1.3%) and high values of fibre count (4000 fibres/g) in the samples corresponding to the late Roman Period (85 cm) and elevated pollen values (7.9%) in the XVIII century (35 cm) but essentially no hemp fibres in the sediment. This suggests cultivation of hemp in the lake vicinity in the Early Modern Period however, no HR. Therefore, we propose the fibre count analysis as a significant additional proxy for investigating HR, especially in the lake sediments with low cannabis type pollen percentages.

The composition of bacterial communities of a hemp rettery was significantly different to the microbiome of the recovered lake. We observed enrichment in the abundance of Chloroflexi, Planctomycetes and Bacteroidetes in the ‘HR’ group, and Proteobacteria and Actinobacteria in the ‘nHR’ group. The increase in Actinobacteria abundance has been associated with the meso-/oligotrophic lakes, suggesting the recovery of LS after the episodes of HR^32^. Most Actinobacteria are aerobic and can form dormant spores under low nutrient conditions. Although many taxa are capable of plant polysaccharides degradation, the abundance of Actinomycetes usually decreases with decreasing oxygen levels^33^, as observed in LS. Proteobacteria are generally abundant in lakes and lake sediments and range from 20% to even 80%^34^. They were reported to be characteristic of both oligotrophic^32,35^ and eutrophic lakes^34^ which is most probably associated with a great ecological richness within this phylum^33^. Proteobacteria, including Betaproteobacteria, Deltaproteobacteria and Gammaproteobacteria, were more predominant in the ‘nHR’ group. These taxa consist of diverse, mostly aerobic bacteria presenting rich catabolic capabilities and important for the biogeochemical cycling of elements^36^. Especially the Betaproteobacteria orders play an important role in nitrification^37^, denitrification^38^ and nitrogen fixing^39^. Although many different proteobacteria are capable of anaerobic organic matter utilisation, also of plant polysaccharide origin^36^, upon the conditions of hypoxia and high trophy associated with HR, the proteobacteria classes did not shift towards anaerobic and/or fermentative orders but instead, became less abundant.

Chloroflexi, Planctomycetes and Bacteroidetes are gram-negative nonproteobacteria capable of anaerobic metabolism. Chloroflexi, the second most represented bacterial phylum in LS, became enriched during HR. This group is relatively understudied, with Dehalococcoidia’s role in halogen cycling and dechlorination being best understood^40^. As more metagenome-assembled genomes (MAGs) of Chloroflexi become available, their role in sediment carbon cycling including fermentation and anaerobic carbon fixation is being recognised. For example, the reductive Acetyl-CoA pathway recently described in the uncultured sediment-associated Chloroflexi^41^ was partially inferred in the ‘HR’ group possibly suggesting their functional role. Planctomycetes constitute another understudied bacterial taxon and have been shown to be more abundant in high trophy lakes^42,43^. Like proteobacteria, Planctomycetes exhibit very diversified metabolism including chemoheterotrophic aerobes and facultative anaerobes and are capable of anammox type reactions, as well as complex carbohydrate fermentation^32,33^. Bacteroidetes were identified to be associated with the dew-retting of hemp^14^. They take part in the degradation of complex biopolymers, including cellulose and pectin, and increase abundance in response to elevated dissolved organic carbon and after cyanobacterial blooms^33^. The role of Chloroflexi, Planctomycetes and Bacteroidetes in the HR process was further reiterated as they were identified to be involved in the inferred cellulose and pectin hydrolysis pathways. Such shifts in bacterial communities towards anaerobic or facultatively anaerobic phyla capable of plant polysaccharides utilisation indicate retting-induced hypoxia of the lake and show bacterial adaptations to the conditions of low oxygen and high plant organic matter. This was supported by the set of environmental factors which best explained the distribution of taxonomic data and included HR, forestation, fibre count and carbonates. Hence, the overrepresented taxa in the ‘HR’ group indeed encompassed more polysaccharides utilising bacteria with no or low oxygen requirements while the overrepresented taxa in the ‘nHR’ group constituted a balanced ecological system.

During the episodes of HR, the sources and ratios of the allochthonous vs autochthonous organic matter were biased. The main source of autochthonous organic matter in lakes is usually phytoplankton and aquatic macrophytes while allochthonous organic matter originates from the catchment, especially terrestrial plants. These origin differences affect the sediment carbon to nitrogen ratio (C/N) as terrestrial plants are rich in polysaccharides and phytoplankton exhibits higher protein content^44^. During HR, an additional source of allochthonous organic matter was introduced into the lake in the form of hemp straws disturbing the C/N which affected the microbiome metabolism. In the ‘nHR’ bacterial community the overrepresented reads were classified to diverse types of metabolism including different nitrogen and sulfur metabolic pathways, together with carbon metabolism, while the functional profile of the ‘HR’ group was dominated by the carbon cycle, especially the anaerobic pathways.

The functional structures of the two groups were significantly different, however, characteristic of low oxygen conditions of the benthic environment. LS is a dimictic, thermally stratified lake exhibiting temperature and density dependent water gradient. Consequently, in the summer and winter months, the deepest strata may become oxygen poor^45^. In LS, under the conditions of oligotrophy and in the absence of HR, light could reach deeper layers of the lake (increased euphotic zone) allowing growth of anoxygenic phototrophic bacteria capable of sulfur metabolism, as shown by the taxonomy and metabolic potential analyses. The dissimilatory sulfate reduction and oxidation, as well as SOX metabolic pathways, which depend on light and inorganic sulfur compounds availability, were overrepresented in the ‘nHR’ group and classified predominantly to different Proteobacteria^46^. During the episodes of HR, trophy increased significantly resulting in the decreased euphotic zone, likely rendering the phototrophic sulfur bacteria dependent metabolism very limited or even impossible. As dissimilatory sulfate reduction is the major anaerobic process of organic carbon mineralisation^47^, disturbed sulfur cycling can affect microbial metabolism. Indeed, eutrophication and lake shallowing were shown to influence microbial taxonomy and functionality and thus, redox chemistry and sulfur metabolism in lake sediments^48^. During the episodes of reduced anthropopressure, the sediment microbiome also exhibited increased potential for dissimilatory nitrate reduction and denitrification as well as, to some degree, nitrification. The first two processes are anaerobic and characteristic of the long-term anaerobic habitats such as freshwater sediments (dissimilatory nitrate reduction) or habitats of transient oxygen levels such as oligotrophic sediments or soils (denitrification). Both pathways take part in the electron transport in the absence of oxygen^49^, however, were not reconstructed in the ‘HR’ group, most probably because they depend on available nitrate as an electron acceptor, which is depleted in shallow eutrophic lakes^48^. The availability of sulfate and nitrate is also dependent on pH and amount of organic carbon^50^, both of which varied upon HR.

Anaerobic carbon fixation was by far the most abundant pathway of carbon metabolism in both groups which is usual for the oxygen low conditions of the lake sediments. Under the conditions of an increased trophy, the microbiome of a hemp rettery showed higher potential for anaerobic carbon fixation and fermentation, while the microbiomes in the ‘nHR’ group were more abundant in marker genes of the aerobic pathways (aerobic carbon fixation and CO oxidation). The utilisation of complex polysaccharides of the allochthonous origin has been linked to the oligo-mesotrophic lakes in which the autochthonous carbon sources are limited^42^. In LS however, the increase of trophic status during HR was a result of introducing large amounts of allochthonous carbon—hemp stems rich in complex plant polysaccharides. The microbiome of the rettery was capable of the utilisation of this carbon source as near complete cellulose and pectin degradation pathways were overrepresented in this group. This functional characteristic of the rettery microbiome was also shown experimentally^51^. Thus, it can be concluded that over the centuries the source and amount of organic carbon in LS were one of the drivers of the microbiome fluctuations, both taxonomic and functional. In contrast to nitrogen and sulfur metabolism, complex plant polysaccharides were degraded predominantly by Chloroflexi, Planctomycetes, Bacteroidetes and Clostridia demonstrating the considerable metabolic potential and environmental significance of these still understudied bacterial taxa^33^.

## Conclusions

Using the NGS and paleo-ecological data we were able to examine the microbiome structure and function, as well as trophy changes in LS, the site of an ancient hemp rettery. This provided an excellent opportunity to investigate the microbial response to a well-defined stressor over several centuries. To the authors’ best knowledge, the lake trophy and microbiome of a historical hemp retting site have not been described before. We showed that the increased allochthonous carbon supply (hemp stems) to the lake correlated with the conditions of elevated trophy and low dissolved oxygen, as well as shifts in the microbial taxonomy and functionality. During HR, the lake microbiome was dominated by the complex polysaccharide utilising bacteria capable of anaerobic and/or facultatively anaerobic growth, while upon cessation of HR practices both lake trophy and microbiome recovered.

## Materials and methods

### Lake bottom core collection and sub-sampling

The LS-C19 sediment core of 98 cm was sampled in January 2019 from the central part of the frozen lake (51°18′17.06″N, 23°21′56.22″E) at a water depth of 8.00 m, with Ø90 mm UWITEC gravity corer. Immediately after collection the core was wrapped in non-transparent black foil and transported for sub-sampling to a laboratory where no previous work with environmental samples had been performed. 1 cm thick samples in the form of disks were collected resulting in a total of 98 samples. The layers were divided with a stainless steel cutting blade and samples for DNA extraction were collected from the middle of each cut disk with a stainless-steel spoon, ensuring an approximately 5 cm margin to the edge of the disk. All stainless-steel utensils were cleaned with distilled water and 70% ethanol between uses. Samples for DNA extraction were immediately transferred into plastic bags, flash frozen in liquid nitrogen and stored at − 80 °C. The remaining material was transferred into plastic bags and stored at 4 °C until further processing.

### Radiocarbon dating and age-depth modelling

The composite age-depth model was based upon ^210^Pb and radiocarbon AMS dating (Supp Table 5). Data was obtained from 3 parallel profiles: JS-A (^210^Pb, unpublished), JS-c^16^ (1 AMS ^14^C) and LS-C19 (3 AMS ^14^C), which were precisely correlated based on palynological, LOI_550_, LOI_950_ and subfossil Cladocera data (Supp Fig. 3, Supp Table 1).

The ^14^C measurements were performed at the Poznań Radiocarbon Laboratory using the Accelerator Mass Spectrometry. The ^14^C dates were calibrated using OxCal 4.4.2 software^52^ with the application of the IntCal20 calibration curve^53^. For age-depth modelling the P_Sequence algorithm of OxCal was used^52,54^ with the parameters: k0 = 0.2, log10(k/k0) = 2, and interpolation = 1 cm.

The sediment sequence of JS-A core was dated by ^210^Pb method, at the Laboratory of Institute of Geological Sciences, Polish Academy of Science in Warsaw. For ^210^Pb analysis, 3 cm^3^ of sediment was taken from each level. For all sediment samples, bulk density and water content were determined. The samples were dried at 105 °C to constant weight and homogenized in an agate mortar. The ^210^Pb activity was determined indirectly by alpha-spectrometry measurement of ^210^Po (Εα = 5.31 MeV, T1/2 = 138 days) activity in 0.1–0.9 g sub-samples^55^. Polonium was extracted from the samples using concentrated hydrochloric and nitric acids and deposited on silver disks^55^. For the complete removal of organic matter, 30% perhydrol was used. The activity of ^210^Po and ^208^Po was measured via an alfa OCTETE PC (EG&G ORTEC) in 12 samples. A known amount of ^208^Po (Εα = 5.11 MeV) was added to the weighted sample as an internal control, and the constant rate of unsupported ^210^Pb supply model (CRS) was used to calculate the sediment age^56^. Supported ^210^Pb was determined by measurements of old sediments (older than 150–200 years) that contain no allochthonous ^210^Pb, assuming constant activity of authigenic ^210^Pb along the sediment column. For samples over the extent of the dating method, age was determined by extrapolation of the sedimentation rate of the lowermost samples. An age-depth function was calculated using the randomization method and fitted by means of the LOESS procedure^57^.

### Loss on ignition (LOI)

Contents of the organic matter and carbonates in the sediments were determined by a sequential loss-on-ignition method based on combustion of dry, homogenized sediment in 550 °C/4 h (organic matter) and 950 °C/2 h (carbonates)^58^ (Supp Data 1).

### Palynological analysis

The selected samples (1 cm^3^ of sediment) were macerated using the standard method of Erdman’s acetolysis. Carbohydrates were removed with 10% HCl and the mineral fraction with 40% HF^59^. Pollen spectra were counted on at least two microscopic slides 18 × 18 mm. From 400 up to 500 grains were counted per single sample. Pollen concentration in all examined samples was high (362,000–1,298,000 per cm^3^). The cumulative sum used for percentage calculations was the sum of tree and shrub pollen (arboreal pollen—AP), and of herb pollen (non-arboreal pollen—NAP), excluding the pollen of aquatic and reed-swamp plants, *Pteridophyta* and *Bryophyta* spores, as well as *Pediastrum* and *Botryococcus* algae colonies. The percentage of AP was used as a forestation proxy, whereas the NAP was further divided into proxies describing types of anthropogenic influence^60^. These included pollens of different cereals (*Secale cereale*, *Triticum* sp., *Fagopyrum* sp., *Linum usitatissimum* and others assigned as Cerealia undiff.) and hemp (*Cannabis* sp.) describing cultivation of these crops. Pollen of *Plantago lanceolata* was used here as a proxy for pasture and pollens of segetal and ruderal weeds including *Centaurea cyanus*, *Convolvulus arvensis*, *Spergula arvensis*, *Scleranthus annuus*, *Artemisia* sp., *Chenopodiaceae*, *Brassicaceae*, *Urtica* and *Rumex acetosella* were used as indicators of anthropogenic impact (Supp Fig. 2, Supp Table 3).

### Plant macrofossil material (hemp fibres) identification and quantification

The count of plant macrofossil material (hemp fibres) was estimated. 100 µL of wet sediment from each layer was weighed and diluted tenfold with distilled water. Thirty aliquots of 10 µL per sample were prepared and diluted further to obtain sufficient visibility of the individual sediment components in a petri dish. The quantity of fibres was counted in each aliquot and the number of fibres per sample was expressed as an arithmetic mean (n = 30). This was then converted into a number of fibres per 1 g of wet sediment.

The identification of hemp fibres in the 8 samples (from the depths 35, 55, 63, 65, 67, 76, 85 and 93) was performed by application of polarised light microscopy (PLM) with a modified Herzog test^22^.

### Diatoms

Diatom samples were prepared using standard techniques introduced by Battarbee^61^. In total, 18 samples were treated with 10% HCl to remove carbonates, washed with distilled water, and treated with 30% H_2_O_2_ in a water bath to remove organic matter. Diatoms were mounted in Naphrax on permanent microscopic slides. Species identification was based on literature^62–68^. In each sample, about 500 diatom frustules were counted to estimate the relative abundance of individual taxa. The taxonomic nomenclature was based on AlgalBase^69^. The list of dominant diatom taxa and diatoms percentage diagrams of LS-C19 core are provided in Supp Table 2 and Supp Fig. 4 respectively.

### Cladocera

Samples of 1 cm^3^ were prepared according to a slightly modified standard procedure^70^. Each sample was heated in 10% KOH until boiling, with a subsequent manual stirring. The residue was washed with distilled water through a 33-µm mesh sieve, treated with 10% HCl, washed again, transferred into a scaled tube and topped up to 10 ml volume. Prior to counting, the remains were stained with safranin. The samples were analysed under a compound light microscope with a 100–400 × magnification. All the remains from each sample were enumerated. A minimum of 200 remains of Cladocera were counted in each sample. The most abundant body part of each taxon was chosen to represent the number of individuals. The percentage of planktonic and littoral taxa (P/L ratio) was used as a lake water level inference. Cladocera based trophy inference was estimated based on the ecological preferences of the examined taxa^71,72^ (Supp Table 1, Supp Fig. 3).

### DNA isolation and sequencing

A total of sixteen samples originating from eight layers (in duplicates), with a mass of two to three grams of frozen sediments were processed for *sed*DNA extraction. The samples were ground in liquid nitrogen using pestle and mortar as well as aluminium oxide as an abrasive and lysis buffer (6 M guanidine hydrochloride, 1% *N*-lauroylsarcosine sodium salt, 50 mM Tris–HCl pH 7.6, 10 mM EDTA, 1% β-mercaptoethanol). Once thawed, the supernatants were collected by centrifugation and incubated with 6 mg proteinase K for 2 h at 55 °C. The samples were further purified using the phenol:chloroform extraction method, followed by overnight ethanol precipitation at − 20 °C. DNA samples were then purified with a commercially available Anti-Inhibitor Kit (A&A Biotechnology) to remove PCR inhibitors and were then treated with additional proteinase K digestion (0,6 mg for 20 min at 55 °C). Next, the samples were purified using Zymo DNA Clean and Concentrator-25 kit and stored at − 20 °C. After that, we performed a DNA repair step using a NEBNext^®^ FFPE DNA Repair Mix, followed by an additional clean-up step (Zymo DNA Clean and Concentrator-25 kit) and storage at − 80 °C. This resulted in the absence of visible degradation, as well as improved 260/280 and 260/230 ratios (as measured by NanoDrop). In order to minimise contamination risk, all utensils including pestles, mortars, pipettes, as well as laboratory benches were routinely cleaned with laboratory grade disinfectant removing DNases, RNases, residual nucleic acids and inactivating viruses and bacteria. A designated laminar flow hood for PCR work was sterilised with UV light before each use. Filtered pipette tips were used for *sed*DNA work. Personal protective equipment was used at all times.

Library preparation and sequencing was performed by Genomed S.A. (Warsaw, Poland). For library preparation NEBNext Ultra DNA Library Prep Kit for Illumina (New England Biolabs, E7645L) was used, according to the manufacturer’s guidelines. Sequencing was performed using Illumina HiSeq 4000 PE150.

### Taxonomic and functional assignment of metagenomes

Sequencing of 16 samples obtained from eight sediment layers resulted in an average of 134.37 million high-quality reads. A total of ~ 314 Gb data was obtained with 147 bp of mean read size distributions for all paired-end fastq files (Supp Data 2a). The quality of data received from the sequencing company was checked with FastQC^73^ software. All samples were pre-filtered and adapters were removed by the sequencing company. FastQC did not report any file with adapters content greater than 0.1%. The samples also met the quality criteria (mean quality score > 35). Filtering was applied using reformat.sh^74^ tool to retain only sequences over 100 nucleotides. The high-quality filtered paired-end reads were assembled into contigs using megahit v1.2.9^75^ with ‘meta-large’ preset which resulted in a total of 2,368,565 to 4,560,710 contigs with 28,732,753,935 total number of bases (N50 values of 556–650 bp). The resulting files were mapped to the entire non-redundant protein sequence database (NCBI NR, accessed on 20.01.2021)^76^ using DIAMOND v. 2.0.6.144^77^. The following parameters were used: --long-reads, block size 5 (-b 5), index chunks 1 (-c 1), the number of CPU threads 28 (-p 28), and the output file as DAA (--outfmt 100), the other parameters remained at default settings. The resulting DAA files were then meganized using the daa-meganizer^78^ tool with the latest available mapping file for NCBI-nr accessions to taxonomic and functional classes (NCBI NR^76^, eggNOG^79^, SEED^80^)—megan-map-Jul2020-2.db. Data from all samples prepared in this way were loaded into the MEGAN v. 6.20.16^78^ software. Using a graphical interface, plots showing the taxonomic composition of individual samples, tables containing all detected taxonomies, unweighted pair group method with arithmetic mean dendrograms (UPGMA), as well as Bray–Curtis dissimilarity matrices based on the data assigned to the bacteria, were obtained.

The SqeezeMeta v. 1.3.0^81^ pipeline in sequential mode was used for functional classification. Contigs obtained at an earlier stage were used (-extassembly option). The key steps of the pipeline consisted of gene prediction using Prodigal^82^ and similarity search to the GenBank^83^, eggNOG^79^ and KEGG^84^ databases using DIAMOND^77^. Prediction of ORF resulted in 3.24–6.04 million of CDS per sample and between 29.73% and 37.85% of CDS per sample were assigned to KEGG Orthology (KO) (Supp Data 2b,c). The data obtained in this way was then loaded into R v. 4.0.4^85^ and, using the SQMtools^86^ library (function loadSQM), only data taxonomically assigned to the bacterial group was selected for further analysis (function subsetTax). Tables containing the abundance and TPM (transcript per million) of each KEGG Orthologs (KOs) for each sample were exported. The TPM of carbon metabolic marker genes (KOs accession numbers) identified in previous studies^26,32^ (Supp Table 4) were compared and presented as a heatmap with pheatmap function of the pheatmap^87^ R package.

### Statistical analysis

Data normalisation for all detected taxonomies was performed in MEGAN^78^ in sample comparison mode (normalisation to the smallest sample size). The Bray–Curtis dissimilarity matrix of the taxonomic data was also constructed in MEGAN. For all samples, bacterial KOs abundances were normalised to TPM values by SqueezeMeta library and were exported using SQMtools library in R. Bray–Curtis dissimilarity matrix was calculated with vegdist function from the obtained normalised bacterial KOs. The normalisation of the environmental data was performed with a function scale from the R base package. The Euclidean distance matrix was calculated for the normalised environmental data using the dist function from the stats R package. All this data was used for the following statistical analyses. Unweighted pair group method with arithmetic mean (UPGMA) clustering analysis and principal component analysis (PCA) were used to display and compare the patterns of taxonomic structure and functional characteristics of the bacterial communities. PCA plots were computed in Statistical Analysis of Metagenomic Profiles (STAMP)^88^ for taxonomy data and all bacterial KOs. UPGMA dendrogram for the taxonomy data was prepared in MEGAN, and for the functional data (all bacterial KOs) was plotted via hclust function from the stats package in R. The significant variance (p < 0.01) between ‘HR’ and ‘nHR’ groups was assessed by permutational multivariate analysis of variance (PERMANOVA) using adonis2 function in the vegan^89^ package. Two group statistical testing was carried out using two-sided Welch's *t* test in STAMP with Welch's inverted confidence (0.95) interval method. A percentile bootstrapping method (1000 replications) was used to estimate confidence intervals, and the false discovery rate (FDR) in multiple testing was corrected with the Benjamini–Hochberg FDR method. Differentially abundant KOs in the ‘HR’ and ‘nHR’ groups, identified by STAMP (corrected p-value < 0.05) were used in MinPath (Minimal set of Pathways)^25^ to reconstruct pathways. The correlation between the overall taxonomic and functional composition was calculated by the Mantel test (9999 permutations, Pearson’s correlation method). Correlation analyses between all environmental factors and the abundances of the taxonomies or functional categories were performed using the bioenv function from the vegan package using Pearson's correlation method. The mantel function was used to calculate empirical p-values, which were corrected for multiple testing using the p.adjust function from the R stats library (Benjamini–Hochberg FDR, BH).

Carbon metabolic marker genes KOs that are differentially segregated across the two groups (‘HR’ vs ‘nHR’) were identified by random forest analysis with Boruta^90^ feature selection (Boruta function in R package Boruta, maxRuns = 9999, mean importance > 4). To infer the genetic potential of carbon metabolic processes, the relative abundance of selected marker genes was used as described elsewhere^26,32^. The individual processes were then normalised to 100% of the total carbon metabolism and their statistical significance was checked using the Wilcox test in R (function wilcox.test in stats package). The data was plotted with a box and whisker plot using the ggplot function of ggplot2^91^ library in R. All the above statistical analyses were performed using R version 4.0.4 and STAMP version v2.1.3.

## Supplementary Information

Supplementary Legends.

Dataset S1.

Dataset S2.

Dataset S3.

Dataset S4.

Dataset S5.

Supplementary Information.

## Data Availability

The cleaned data was deposited in the European Nucleotide Archive (ENA: PRJEB36143, PRJEB39351).
