# Supplementary material for: Lake microbiome and trophy fluctuations of the ancient hemp rettery
Source: Sci Rep. 2022 May 25;12:8846. doi: 10.1038/s41598-022-12761-w (PMC9132974; doi:10.1038/s41598-022-12761-w)
Supplement: Supplementary file 1 — Supplementary Legends. [file 41598_2022_12761_MOESM1_ESM.docx]

**Supplementary Data:**

**SuppData 1.** Loss on ignition raw data.

**SuppData 2.** (**a**) Summary of metagenomic sequencing statistics. (**b**) Summary of assembly. (**c**) Summary of CDS prediction and KEGG assignment.

**SuppData 3.** (**a**) STAMP results on phyla data. (**b**) STAMP results on Class-Subclass data.

**SuppData 4.** STAMP results on functional KOs.

**SuppData 5.** minPath results for ‘nHR’ and ‘HR’ groups.
