# Supplementary material for: Lake microbiome and trophy fluctuations of the ancient hemp rettery
Source: Sci Rep. 2022 May 25;12:8846. doi: 10.1038/s41598-022-12761-w (PMC9132974; doi:10.1038/s41598-022-12761-w)
Supplement: Supplementary file 7 — Supplementary Information. [file 41598_2022_12761_MOESM7_ESM.pdf]

Supplementary information for

Lake Microbiome and Trophy Fluctuations of the Ancient Hemp Rettery

Olga Iwańska[1,2,+], Przemysław Latoch[1,3,+], Magdalena Suchora[4,5], Irena A. Pidek[4], Miłosz Huber[4], Iwona Bubak[6], Natalia Kopik[1,2], Mariia Kovalenko[1,2], Michał Gąsiorowski[7], Jean-Paul Armache[8], Agata L Starosta[1,2,*]

[1] ECOTECH-Complex and Institute of Biological Sciences, Maria Curie-Sklodowska University, Lublin, Poland
[2] Institute of Biochemistry and Biophysics, Polish Academy of Science, Warsaw, Poland
[3] Polish-Japanese Academy of Information Technology, Warsaw, Poland
[4] Institute of Earth and Environmental Sciences, Maria Curie-Sklodowska University, Lublin, Poland
[5] ECOTECH-Complex, Maria Curie-Sklodowska University, Lublin, Poland
[6] Department of Hydrology, Institute of Geography, University of Gdansk, Gdansk, Poland
[7] Institute of Geological Sciences Polish Academy of Science, Warsaw, Poland
[8] Department of Biochemistry and Molecular Biology and the Huck Institute of Life Sciences, Pennsylvania State University, Pennsylvania, United States

[+] contributed equally.
* corresponding author:
Agata L Starosta agata.starosta@gmail.com, agata.starosta@ibb.waw.pl

This supplemental material includes:

Supplemental Figure 1-6

Supplemental Table 1-5

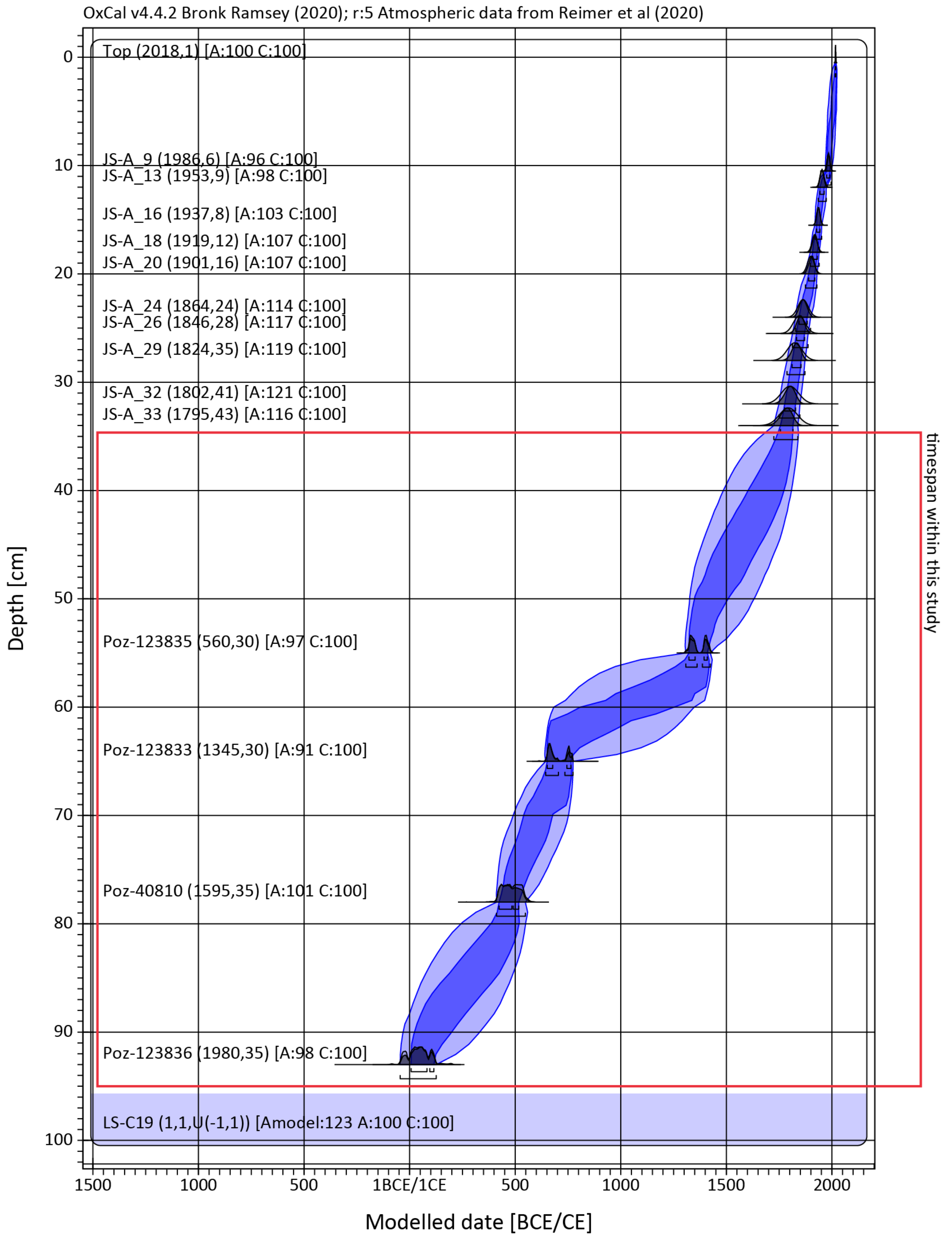

SuppFig 1. Age-depth model – constructed by MS with OxCal 4.4 software (https://c14.arch.ox.ac.uk/oxcal.html).

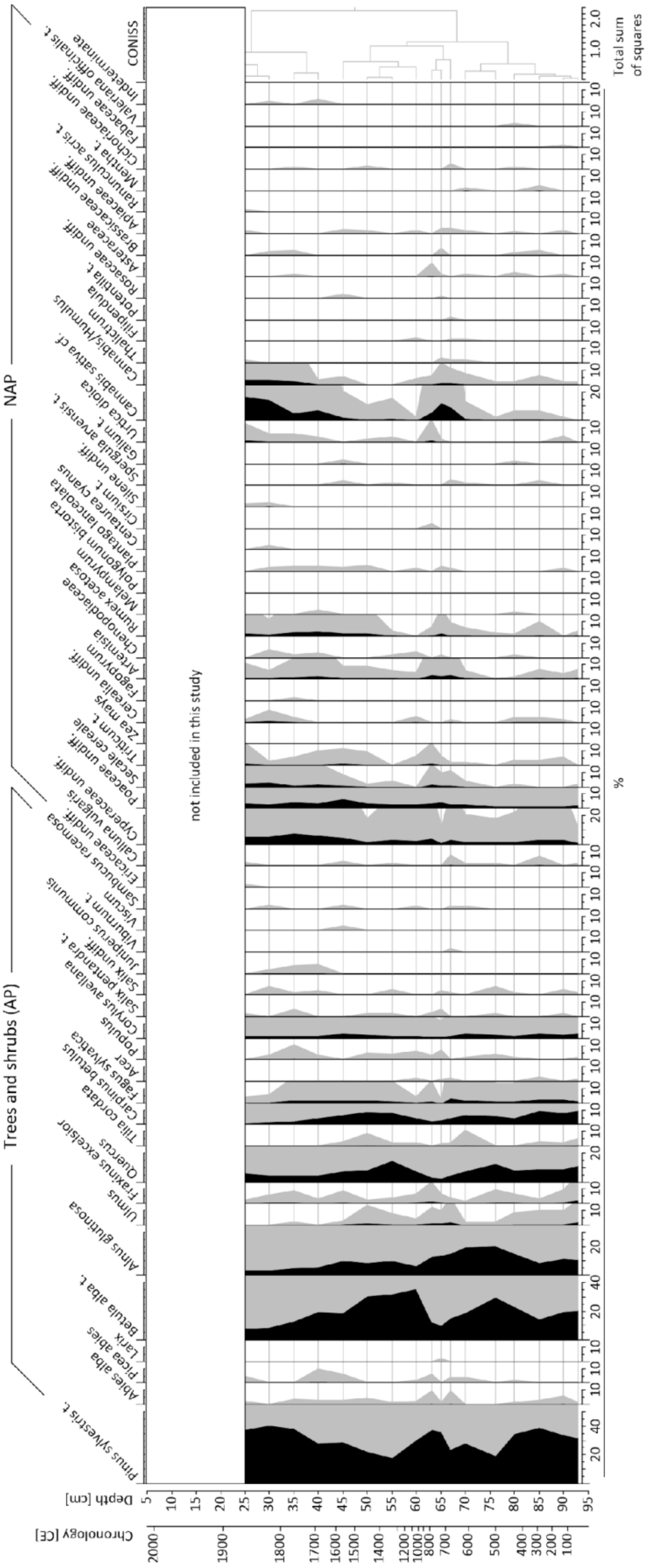

SuppFig 2. Pollen percentage diagram of LS-C19 core – drawn by MS with Tilia software (https://www.tiliait.com) based on data provided by IAP.

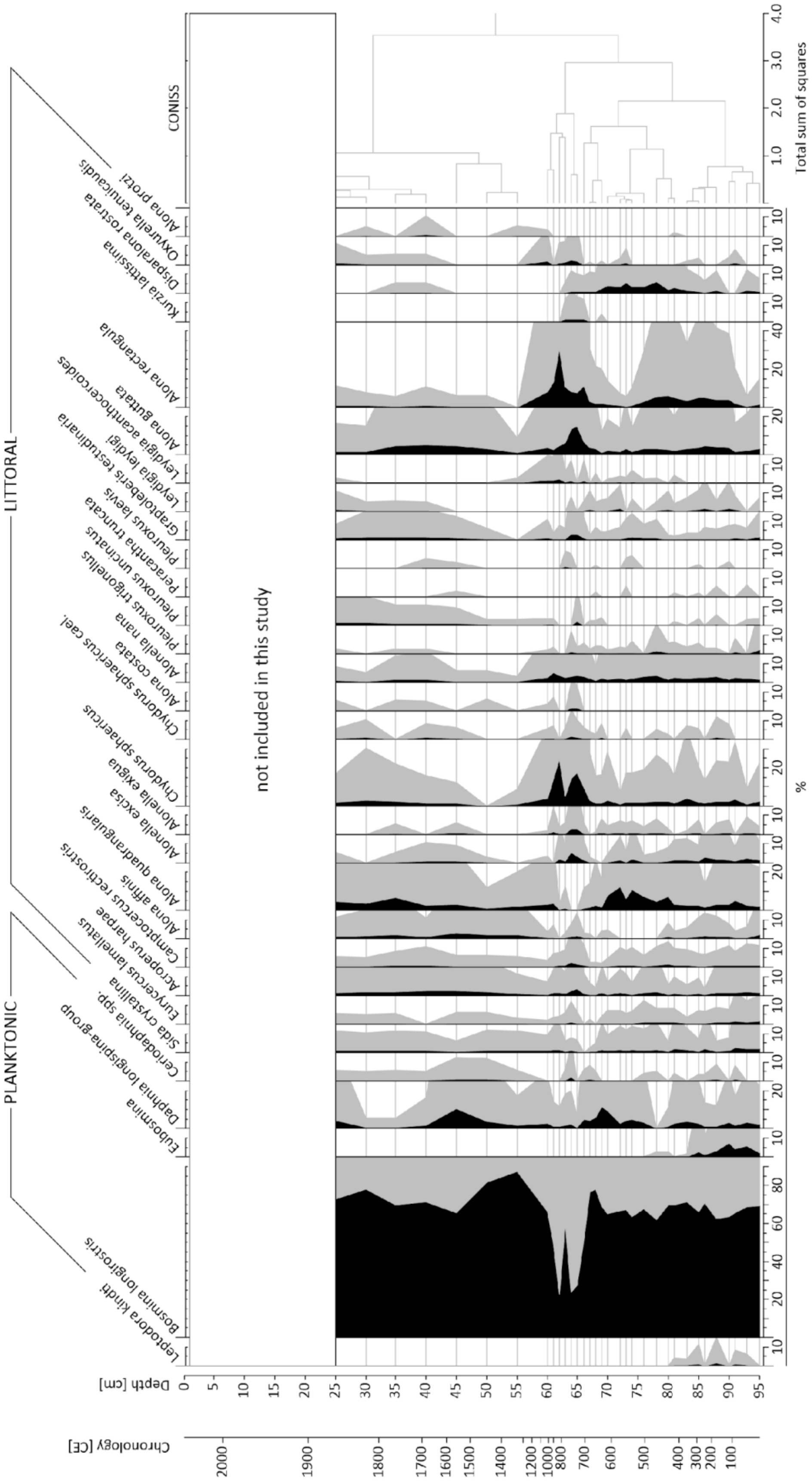

SuppFig 3. Subfossil Cladocera percentage diagram of LS-C19 core – drawn by MS with Tilia software (https://www.tiliait.com), based on our own data.

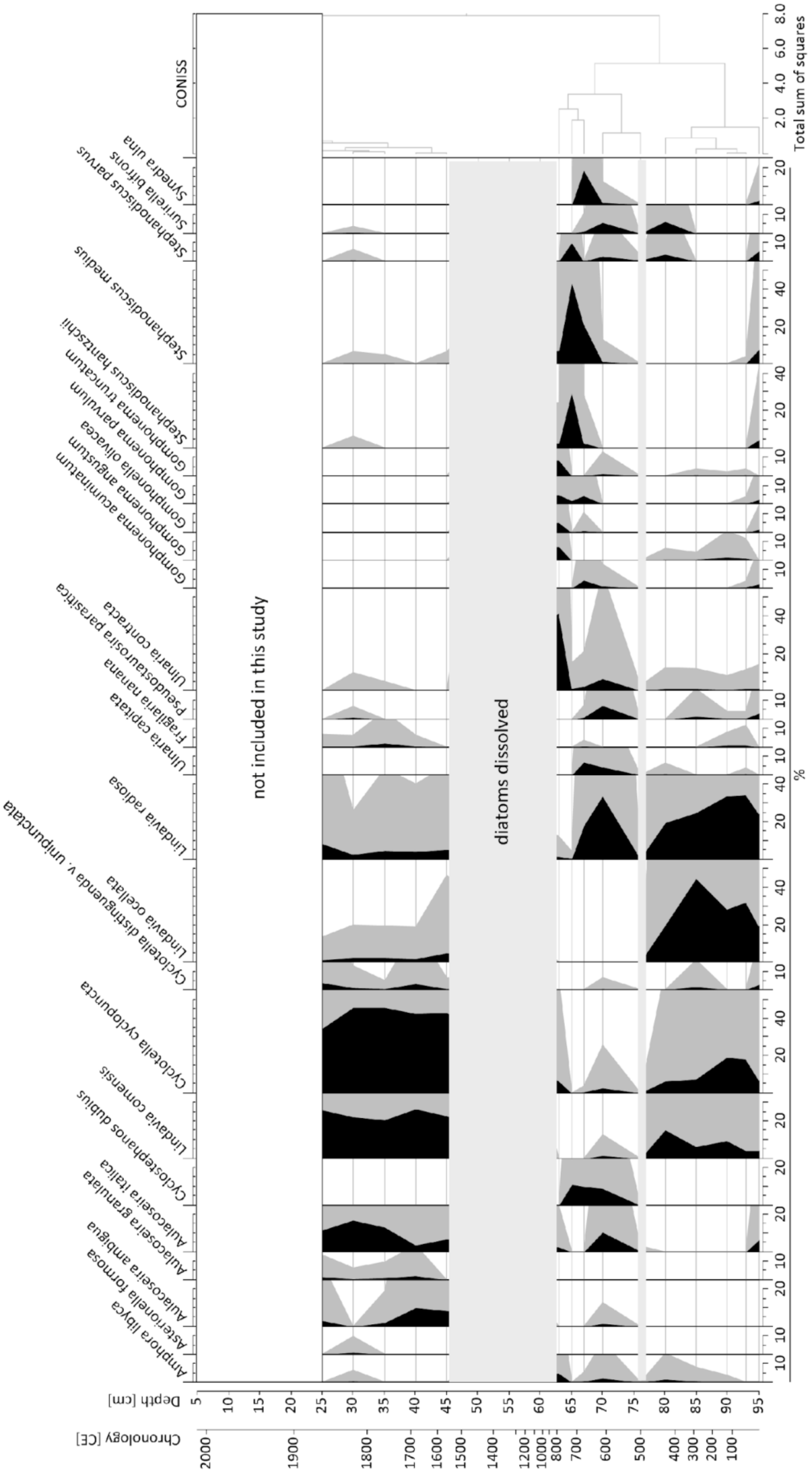

SuppFig 4. Subfossil Dominant Diatoms percentage diagram of LS-C19 core – drawn by MS in Tilia software (https://www.tiliait.com) based on data provided by IB.

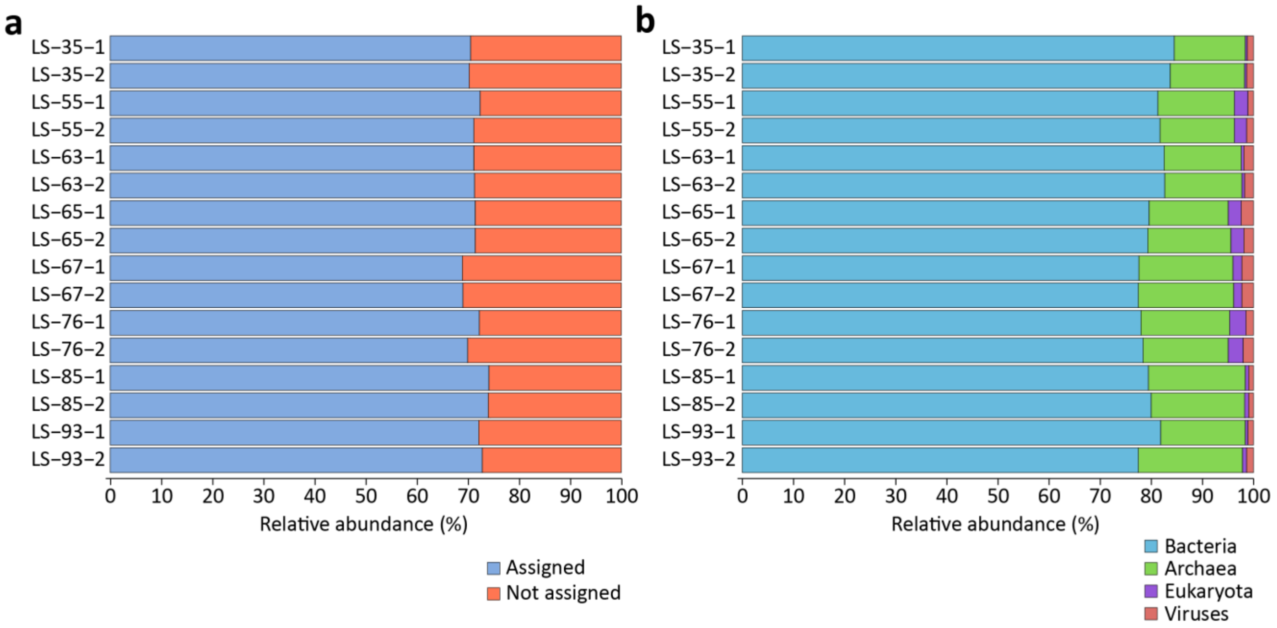

SuppFig 5. (**a**) Taxonomic assignment of sequences per sample. (**b**) Domain assignment per sample.

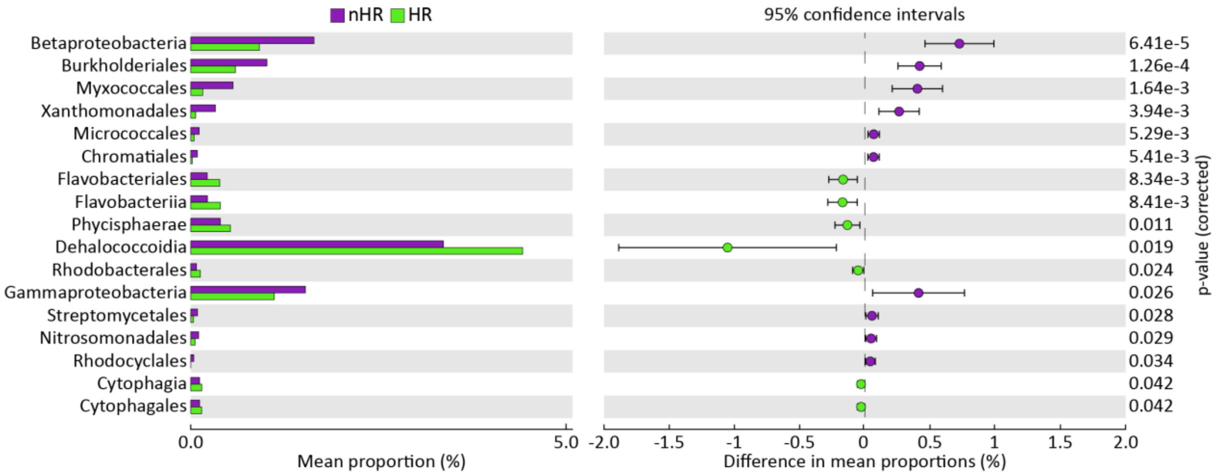

SuppFig 6. Extended error bar plot showing significant differences ($p<0.05$) in the taxa abundances at the class-subclass level for 'HR' (green) vs 'nHR' (purple) groups, calculated with two-sided Welch's t-test (STAMP).

SuppTab 1. List of Cladocera taxa recorded in LS-C19 core and their ecological preferences according to Flössner (2000) and Bjerring *et al.* (2009), Błędzki and Rybak (2010).

taxa	habitat	
	**planktonic/littoral**	**benthic/vegetation**
Leptodora kindti (Focke, 1844)	planktonic	-
Bosmina longirostris (O.F. Muller 1785)	planktonic	-
Eubosmina (*E. coregoni* Baird, 1857 & *E. longispina* Leydig, 1860)	planktonic	-
Daphnia longispina -group (O.F. Muller 1776)	planktonic	-
Ceriodaphnia sp. (Dana, 1853)	planktonic/littoral	vegetation
Sida crystallina (O.F. Muller, 1776)	planktonic/littoral	vegetation
Eurycercus lamellatus (O.F. Muller, 1776)	littoral	vegetation
Acroperus harpae (Baird, 1834)	littoral	vegetation
Camptocercus rectirostris (Schrödler, 1862)	littoral	vegetation
Alona affinis (Leydig, 1860)	littoral	vegetation
Alona quadrangularis (Baird, 1843)	littoral	benthic/vegetation
Alonella excisa (Fischer, 1854)	littoral	vegetation
Alonella exigua (Lilljeborg, 1853)	littoral	vegetation
Chydorus sphaericus s.l. (O.F. Muller, 1776)	littoral/planktonic (while blue-green algae bloom)	varied
Alona costata (Sars, 1862)	littoral	vegetation
Alonella nana (Baird, 1834)	littoral	vegetation
Pleuroxus trigonellus (O.F. Muller, 1776)	littoral	vegetation
Pleuroxus uncinatus (Baird, 1850)	littoral	vegetation
Peracantha truncata (O.F. Muller, 1785)	littoral	vegetation
Pleuroxus laevis (Sars, 1862)	littoral	vegetation
Graptoleberis testudinaria (Fischer, 1848)	littoral	vegetation
Leydigia leydigi (Schrödler, 1863)	littoral	benthic
Leydigia acanthocercoides (Fischer, 1854)	littoral	vegetation
Alona guttata (Sars 1862)	littoral	vegetation
Alona rectangula (Sars 1861)	littoral	vegetation
Kurzia lattissima (Kurz, 1875)	littoral	vegetation
Disparalona rostrata (Koch, 1841)	littoral	benthic
Oxyurella tenuicaudis (Sars, 1862)	littoral	vegetation
*Alona protzi (*Hartwig 1900)	littoral	benthic/phreatic
Latona setifera (O.F. Műller, 1776)	littoral	benthic
Anchistropus emarginatus (Sars, 1862)	littoral	vegetation
Camptocercus lilljeborgi (Schrodler, 1861)	littoral	vegetation
Camptocercus fennicus (Stenroos, 1898)	littoral	vegetation
Poyphemus pediculus (Linné, 1761)	littoral	vegetation

Cladocera taxa included in trophy inference as high trophy indicators (counted as the percentage among total littoral taxa): *Alona rectangula*, *Chydorus sphaericus*, *Pleuroxus uncinatus*, *Leydigia leydigi*, *Leydigia acanthocercoides*, *Disparalona rostrata*, *Oxyurella tenuicaudis*, *Camptocercus lilljeborgi* and *Graptoleberis testudinaria.*

SuppTab 2. List of dominant diatom taxa recorded in LS-C19 core and their ecological preferences; habitat system by Round (1981), pH system by Hustedt (1937–39), trophic system by Neumann (1932).

**Taxa**	**habitat**	**pH**	**trophy**
Amphora libyca Ehrenberg	benthic	alkaliphilous	oligotraphentic
Asterionella formosa Hassal	planktonic	alkaliphilous	eu-mesotraphentic
Aulacoseira ambigua (Grunow) Simonsen	planktonic	alkaliphilous-neutrophilic	eutraphentic
Aulacoseira granulata (Ehrenberg) Simonsen	planktonic	alkaliphilous	eutraphentic
Aulacoseira italica (Ehrenberg) Simonsen	planktonic	neutrophilic - alkaliphilous	eutraphentic
Cyclostephanos dubius (Fricke) Round	planktonic	alkaliphilous	eutraphentic
Cyclotella cyclopuncta Håkansson & J.R.Carter	planktonic	neutrophilic	oligotraphentic
Cyclotella distinguenda v. unipunctata (Hustedt) Håkansson & J.R.Carter	planktonic	neutrophilic	oligotraphentic
Fragilaria nanana Lange-Bertalot	benthic	neutrophilic	oligotraphentic
Gomphonema acuminatum Ehrenberg	benthic	alkaliphilous-neutrophilic	eu-dystraphentic
Gomphonema angustum Agardh	benthic	neutrophilic	eu-dystraphentic
Gomphonella olivacea (Hornemann) Rabenhorst	benthic	alkaliphilous	eu-dystraphentic
Gomphonema parvulum (Kützing) Kützing	benthic	alkaliphilous-neutrophilic	eu-mesotraphentic
Gomphonema truncatum Ehrenberg	benthic	alkaliphilous	eu-dystraphentic
Lindavia comensis (Pantocsek) Nakov et al.	planktonic	neutrophilic	oligotraphentic
Lindavia ocellata (Pantocsek) Nakov et al.	planktonic	alkaliphilous	oligotraphentic
Lindavia radiosa (Grunow, Lemmermann) De Toni & Forti	planktonic	alkaliphilous	mesotraphentic
Pseudostaurosira parasitica (Smith) Morales	benthic	alkaliphilous	eu-mesotraphentic
Stephanodiscus hantzschii Grunow	planktonic	alkalib- alkaliph	eutraphentic
Stephanodiscus medius Håkansson	planktonic	alkaliphilous	eutraphentic
Stephanodiscus parvus Stoermer & Håkansson	planktonic	alkalibiontic	eutraphentic
Surirella bifrons Ehrenberg	benthic	alkaliphilous	oligotraphentic
Synedra ulna (Nitzsch) Ehrenberg	benthic	alkaliphilous-neutrophilic	eutraphentic
Ulnaria capitata Ehrenberg Compère	benthic	alkaliphilous	eu-mesotraphentic
Ulnaria contracta (Østrup) Morales & Vis	benthic	alkaliphilous	eutraphentic

SuppTab 3. Investigated environmental factors with values for all samples.

Samples description				Environmental factors								
**Name**	**Depth [cm]**	**Age [years]**	**Hemp retting [Yes/No]**	**Cereal* [%]**	**Weeds* [%]**	**Forestation* [%]**	**Pasture* [%]**	**Hemp pollen [%]**	**Carbonates (LOI 950°C × 2,274) [%]**	**Organic carbon (LOI 550°C) [%]**	**Fibres [per 1 g]**	**Trophy** [%]**
LS-35-1	35	1754	N	7.6	9.9	71.4	0.4	7.9	24.73	20.19	400	19.05
LS-35-2	35	1754	N	7.6	9.9	71.4	0.4	7.9	24.73	20.19	400	19.05
LS-55-1	55	1367	N	1.9	5.1	89.4	0	1.5	35.8	11.49	200	10.53
LS-55-2	55	1367	N	1.9	5.1	89.4	0	1.5	35.8	11.49	200	10.53
LS-63-1	63	913	Y	9.4	8.4	76.5	0	6.9	31.1	23.26	5500	48.19
LS-63-2	63	913	Y	9.4	8.4	76.5	0	6.9	31.1	23.26	5500	48.19
LS-65-1	65	710	Y	14.2	10.1	71.8	0.3	14	25.9	30.95	5000	48.6
LS-65-2	65	710	Y	14.2	10.1	71.8	0.3	14	25.86	30.95	5000	48.6
LS-67-1	67	668	Y	10.9	7.8	74.8	0.2	10.6	29.5	22.57	1000	43.88
LS-67-2	67	668	Y	10.9	7.8	74.8	0.2	10.6	29.5	22.57	1000	43.88
LS-76-1	76	521	N	0.2	1.8	95.9	0	0.4	31.74	21.68	100	33.09
LS-76-2	76	521	N	0.2	1.8	95.9	0	0.4	31.74	21.68	100	33.09
LS-85-1	85	274	Y	1.3	4	89.8	0	1.2	32.57	18.85	4000	30.11
LS-85-2	85	274	Y	1.3	4	89.8	0	1.2	32.57	18.85	4000	30.11
LS-93-1	93	42	N	0.2	3.4	94.8	0	0.2	26.2	29.12	300	13.24
LS-93-2	93	42	N	0.2	3.4	94.8	0	0.2	26.2	29.12	300	13.24

***** Cereal, weeds, forestation and pasture are calculated based on pollen percentages of the following species:

Cereal: *Secale cereale*, *Triticum* sp., *Fagopyrum* sp., *Linum usitatissimum*, *Cannabis sativa*, other (Cerealia undiff.)

Weeds: *Centaurea cyanus*, *Convolvulus arvensis*, *Artemisia* sp., *Chenopodiaceae*, *Urtica*, *Brassicaceae*, *Rumex acetosella t.*, *Spergula arvensis*, *Scleranthus annuus*

Forestation: sum of all arboreal pollen

Pasture: *Plantago lanceolata*

******trophy inference was estimated based on the percentage abundance of the examined Cladocera taxa with ecological preferences towards high/low trophy (as listed in SuppTab. 1).

SuppTab 4. Selected carbon metabolic marker genes KOs involved in different pathways of the carbon cycle.

Cycle	KEGG Orthology (KO)	Gene
Anaerobic Carbon Fixation	K01648	ACLY; ATP citrate (pro-S)-lyase [EC:2.3.3.8]
	K00174	korA, oorA, oforA; 2-oxoglutarate/2-oxoacid ferredoxin oxidoreductase subunit alpha [EC:1.2.7.3 1.2.7.11]
	K00175	korB, oorB, oforB; 2-oxoglutarate/2-oxoacid ferredoxin oxidoreductase subunit beta [EC:1.2.7.3 1.2.7.11]
	K00244	frdA; fumarate reductase flavoprotein subunit [EC:1.3.5.4]
	K00194	cdhD, acsD; acetyl-CoA decarbonylase/synthase, CODH/ACS complex subunit delta [EC:2.1.1.245]
	K00197	cdhE, acsC; acetyl-CoA decarbonylase/synthase, CODH/ACS complex subunit gamma [EC:2.1.1.245]
Aerobic Carbon Fixation (Calvin cycle)	K00855	PRK, prkB; phosphoribulokinase [EC:2.7.1.19]
	K01602	rbcS, cbbS; ribulose-bisphosphate carboxylase small chain [EC:4.1.1.39]
Fermentation	K00016	LDH, ldh; L-lactate dehydrogenase [EC:1.1.1.27]
	K00169	porA; pyruvate ferredoxin oxidoreductase alpha subunit [EC:1.2.7.1]
	K00170	porB; pyruvate ferredoxin oxidoreductase beta subunit [EC:1.2.7.1]
Aerobic Respiration	K02274	coxA, ctaD; cytochrome c oxidase subunit I [EC:7.1.1.9]
	K02276	coxC, ctaE; cytochrome c oxidase subunit III [EC:7.1.1.9]
CO Oxidation	K03518	coxS; aerobic carbon-monoxide dehydrogenase small subunit [EC:1.2.5.3]
	K03519	coxM, cutM; aerobic carbon-monoxide dehydrogenase medium subunit [EC:1.2.5.3]
	K03520	coxL, cutL; aerobic carbon-monoxide dehydrogenase large subunit [EC:1.2.5.3]

SuppTab 5. The results of the radiocarbon dating of LS-C19 and JS-c core included in lake-depth modelling.

Depth bsl [cm]	Laboratory code	^{14}C date [^{14}C BP]	Calibrated age - 1σ range [cal BCE/CE] 68.3% probability	Calibrated age - 2σ range [cal BCE/CE] 95.4% probability	Dated material
LS-C19					
55	Poz-123835	560 ± 30 BP	1326 CE (34.9%) 1351 CE<br>1394 CE (33.4%) 1414 CE	1312 CE (48.6%) 1362 CE<br>1386 CE (46.9%) 1428 CE	Terrestrial plant macrofossil
65	Poz-123833	1345 ± 30 BP	650 CE (55.4%) 680 CE<br>747 CE (12.9%) 758 CE	643 CE (68.3%) 705 CE<br>738 CE (27.1%) 774 CE	Terrestrial plant macrofossil (hemp fibres)
93	Poz-123836	1980 ± 35 BP	33 BCE (10.7%) 16 BCE<br>6 CE (54.4%) 78 CE<br>101 CE (3.2%) 106 CE	45 BCE (95.4%) 122 CE	Terrestrial insect macrofossil
JS-c					
852 (depth bwl)<br>102 (depth bsl)	Poz-40810	1595 ± 35 BP	430 CE (10.1%) 442 CE<br>448 CE (25.0%) 480 CE<br>494 CE (33.1%) 536 CE	414 CE (95.4%) 552 CE	Terrestrial plant macrofossil

bwl – below water level

bsl – below sediment level

Comment:
Sediments of LS-C19 core taken with UWITEC sampler (with hammer application) were in the bottommost section more compacted than JS-c, sampled with Instorf corer. Both cores were correlated based on LOI, pollen and Cladocera data.

Supplementary References:

Battarbee, R.W., 1986. Diatom analysis. In: Berglund, B.E. (Ed.), Handbook of Holocene Palaeoecology and Palaeohydrology. John Wiley and Sons. Ltd. London, pp. 527-570.

Hustedt, F. (1937–39). Systematische und ökologische Untersuchungenüber die Diatomeen-flora von Java, Bali und Sumatra III. Die ökologischen Faktoren und ihr Einfluss auf die Diatomeenflora. – Archiv fűr Hydrobiologie Supplement, 15: 131-177, 187-295, 393-506, 638-790, 16: 274-344.

Round, F.E., 1981. The ecology of algae. Cambridge University Press, Cambridge.

Flössner D. (2000) Die Haplopoda und Cladocera (ohne Bosminidae) Mitteleuropas. Backhuys Publishers, Leiden

Bjerring, R., Becares, E., Declerck, S., Gross, E. M., Hansson, L. A., Kairesalo, T., ... & Jeppesen, E. (2009). Subfossil Cladocera in relation to contemporary environmental variables in 54 Pan-European lakes. Freshwater Biology, 54(11), 2401-2417.

Błędzki, L. A., & Rybak, J. I. (2016). Freshwater Crustacean Zooplankton of Europe: Cladocera & Copepoda (Calanoida, Cyclopoida) Key to species identification, with notes on ecology, distribution, methods and introduction to data analysis. Springer.
